# Strategies to overcome physician shortages in northern Ontario: A study of policy implementation over 35 years

**DOI:** 10.1186/1478-4491-6-24

**Published:** 2008-11-11

**Authors:** Raymond W Pong

**Affiliations:** 1 Centre for Rural and Northern Health Research and Northern Ontario School of Medicine, Laurentian University, Sudbury, Ontario, Canada

## Abstract

**Background:**

Shortages and maldistibution of physicians in northern Ontario, Canada, have been a long-standing issue. This study seeks to document, in a chronological manner, the introduction of programmes intended to help solve the problem by the provincial government over a 35-year period and to examine several aspects of policy implementation, using these programmes as a case study.

**Methods:**

A programme analysis approach was adopted to examine each of a broad range of programmes to determine its year of introduction, strategic category, complexity, time frame, and expected outcome. A chronology of programme initiation was constructed, on the basis of which an analysis was done to examine changes in strategies used by the provincial government from 1969 to 2004.

**Results:**

Many programmes were introduced during the study period, which could be grouped into nine strategic categories. The range of policy instruments used became broader in later years. But conspicuous by their absence were programmes of a directive nature. Programmes introduced in more recent years tended to be more complex and were more likely to have a longer time perspective and pay more attention to physician retention. The study also discusses the choice of policy instruments and use of multiple strategies.

**Conclusion:**

The findings suggest that an examination of a policy is incomplete if implementation has not been taken into consideration. The study has revealed a process of trial-and-error experimentation and an accumulation of past experience. The study sheds light on the intricate relationships between policy, policy implementation and use of policy instruments and programmes.

## Background

Geographical maldistribution of health care providers, especially physicians, is a ubiquitous problem, affecting many countries and regions. Physicians tend to congregate in larger cities, leaving many rural areas, small towns and remote communities underserved. In Canada, 9.4% of physicians (2.4% of specialists and 16% of family physicians) practised in rural areas, where slightly over 21% of Canadians lived in 2004 [1]. In their seminal report, *Toward integrated medical resource policies for Canada*, Barer and Stoddard [2] identified maldistribution of physicians as one of five "first tier" (i.e. most critical) problems. Similarly, the Commission on the Future of Health Care in Canada [3] pointed out that "(a)ccess to physicians and specialists varies significantly across the country and some communities do not have access to the most basic health care services because they lack the necessary health care providers" (p. 162).

Severe and persistent maldistribution of physicians is clearly an unacceptable situation, especially in Canada, where there is a national Medicare system, with two of its five basic principles being "accessibility" and "universality". Many strategies have been used to effect a more equitable distribution of physicians. Knowing what influences physicians to work or not to work in rural areas helps us understand why certain strategies are adopted. These include rural background [4-6], family factors including spousal influence [7,8], medical education [9-12], medical practice [13,14], and income [13]. Goertzen [15] has identified four sets of factors that are believed to encourage rural practice: personal interests and background, appropriate medical training, community attributes and working conditions.

This study has two objectives. First, it documents, in a chronological manner, the introduction of programmes designed to alleviate physician shortages in northern Ontario over a 35-year period from 1969 to 2004 and examines changes in the use of policy instruments. This is done by charting the introduction of new programmes, including those subsumed under the Underserviced Area Program (UAP). A related objective is to examine several aspects of policy implementation, using these programmes as a case study. These two objectives are complementary.

This study is predicated on the belief that government-initiated programmes are manifestations of public policies which, quite often, remain implicit or are couched in broad generalities. Whereas policies are statements of ideologies, political agenda, values or government priorities, programmes are means to translate policies into desired outcomes. Between policies and programmes one may also find implicit policy instruments, which are broad strategies used by policy-makers to guide or design programmes. Thus, the nature and characteristics of a programme reflect the policy behind it and the preferred policy instrument. By examining how programmes are introduced, modified or terminated, one could deduce shifting policy perspectives on an issue and the strategies adopted.

Research on policy implementation has traditionally focused on the approaches used, such as "top-down" or "bottom-up", and complexities and challenges facing implementation [16-18]. Moving beyond these typical concerns, this study seeks to examine what policy instruments were used and how they changed over time. It has been said that studies of policy instruments have contributed to a better understanding of Canadian public policy [19]. This study hopes to further this area of research. But, instead of focusing on a single programme, it looks at how a broad array of programmes has been introduced over a 35-year period. Sabatier [18] has criticized American policy implementation research for using a short time frame. A longer time frame is also needed in policy implementation studies in Canada because, as Fooks [20] has observed, "(t)he Canadian health policy culture is not an environment in which rapid change is easily achieved" (p. 131).

This study pays special attention to several aspects of policy implementation. Public policy-making has been seen by some as a rational approach and described by others as a process of "muddling through", involving small, incremental changes [21]. Policy implementation processes may be similarly characterized, since policy formulation and policy implementation often overlap [22]. This study seeks to find out if policy implementation is a rational or an incremental process. It also tries to understand why some policy instruments were chosen, while others were not.

The policy at issue is the Ontario government's stated intention to ensure a sufficient physician supply to serve the population in northern Ontario – a vast territory of about 800,000 sq km with a widely scattered population of about 800,000. It might not be a mere coincidence that the UAP was established in 1969, the very same year when the Ontario Health Insurance Plan (OHIP) – the provincial Medicare programme – was introduced. OHIP was intended to ensure universal access to needed medical and hospital care for all Ontarians regardless of economic means. But removal of financial barriers to health care is meaningless if providers and services are not available or are very difficult to access. Thus, as far back as 1969 (and possibly earlier), the Ontario government saw shortages of health care providers, especially physicians, in northern Ontario as a problem that needed attention and intervention.

While the magnitude of the problem may have changed, the policy goal does not appear to have shifted since 1969. This can be gleaned from various policy declarations over the years contained in speeches given by premiers and ministers of health and government press releases. For instance, in announcing the development of programmes to train family physicians in northern Ontario, a press release from the Office of the Premier [23] in 1990 stated, "A Northern Ontario residency training programme for medical school graduates entering family practice was announced today by Premier David Peterson.... 'This new programme promises to help solve the problem of recruitment and retention of physicians in northern, rural and remote communities,' said Mr. Peterson". In announcing the Free Tuition Program in 2000, a ministry of health news release remarked, "'This initiative will provide financial assistance to medical students and address the needs of rural and northern communities,' (Minister) Witmer said. 'We are working with doctors, medical students and communities to ensure that all Ontarians have access to physician services"' [24].

Lucas [25] examined the availability of physicians in small, single-industry communities in Northern Ontario in 1968, just one year before the launch of the UAP. Of the 240 communities examined, 176 (or 73%) were without a doctor and another 23 with only one doctor. After studying the numbers of physicians in northern Ontario from the 1950s to the 1980s, Anderson and Rosenberg [26] concluded that the UAP had not improved the supply or distribution of physicians in that region. However, a more recent study [27] shows that northern Ontario had an increase of 6.2 full-time-equivalent family physicians (specialists not included) per 100 000 population between 1993/1994 and 2001/2002, whereas all other regions of the province experienced a negative growth. However, it is not the intent of this study to assess the impact of the UAP and other programmes, individually or collectively.

## Methods

Information about programmes to help overcome physician maldistribution, their characteristics and the years of programme introduction was obtained from official documents, programme brochures, web sites and discussions with government officials. A study by Tepper and associates [28] contains a similar list of programmes, which was used to verify information accuracy.

Of all the programmes examined, the most important is the UAP. Initiated in 1969, the UAP is one of the largest and longest-lasting programmes of its kind in North America. It is an interrelated set of programmes funded and, in some cases, administered by the ministry of health and designed to attract health care practitioners, including physicians, to work in northern Ontario. It is the programmes subsumed under the UAP and other programmes with the same objective but not under the UAP umbrella that are of interest to this study. Although the UAP has expanded in more recent years to cover some underserved communities in southern Ontario, the focus of this study is on northern Ontario.

Several criteria were used to decide which programmes were to be included in the study. Programmes must be financially supported by the provincial government, though not necessarily funded or administered by the ministry of health. Federal government initiatives were not included. Similarly, programmes belonging to non-government agencies were excluded because the study is primarily interested in public policy. Also not included were "generic" strategies that did not specifically target northern Ontario, such as Ontario medical school enrolment expansion and fast-tracking of international medical graduates into practice. They might have workforce implications for the north, but they were province-wide programmes and often had a marginal impact on northern Ontario.

Some programmes have evolved over the years. For example, the Northern Health Travel Grants Program has been modified several times with respect to eligibility criteria and subsidy level. The management of some locum tenens programmes has shifted from the ministry of health to other agencies. Such operational changes have been ignored, as this study is about changes in policy instruments used and not about programme administration.

The programmes included in this study can be analysed not only in terms of the types of strategy used, but also in terms of the time frame of expected outcomes, degree of programme complexity and outcome objectives. The methodology used is programme analysis: the nature of each programme was examined to determine its strategic category, time frame, and so on.

Policy researchers have suggested different ways to classify policy instruments [29-31]. The strategies used to overcome geographical maldistribution of physicians can also be categorized in different ways. For instance, Crandall and colleagues [32] have proposed a four-category classification: affinity, economic incentive, practice characteristic and indenture models. Similarly, Barer and Wood [33] have suggested four categories: regulatory/administrative, educational, financial and laissez-faire strategies. But these and similar classification schemes are too broad and not sufficiently discriminating to allow differentiation between programmes or detection of more subtle changes in the use of policy tools. An in-depth examination of changes in policy implementation requires a more elaborate categorization system. Following an examination of the objectives, programme guidelines and specifics of each of the included programmes, the following types of policy instrument were identified:

1. *Financial incentives*: Providing incentives to medical students or physicians willing to work in northern Ontario.

2. *Physician recruitment*: "Marketing" northern Ontario to physicians.

3. *Alternative providers*: Using non-physician practitioners such as nurse practitioners where physicians are not available.

4. *Rural medical education/training*: Training physicians in rural or northern areas.

5. *Medical practice support*: Making northern practice less onerous in order to enhance its attractiveness.

6. *Service outreach*: Bringing services to areas where they are not locally available.

7. *Patient travel assistance*: Providing financial assistance to patients who have to travel long distances to access medical care.

8. *Telemedicine*: Linking patients and physicians via telecommunications technology.

9. *Research*: Using research to support rural health workforce planning.

The next step was to sort each programme into one of the nine categories. A chronology of programme initiation was then constructed, based on the year in which a programme was first introduced. Some programmes may have a long gestation period. For instance, the establishment of the Northern Ontario School of Medicine (NOSM) was officially announced in 2001, but the first cohort of students was not admitted until 2005. Typically, the year when a new programme started operation was chosen as the initiation year. In the case of the NOSM, 2002 was chosen because the Founding Dean was appointed in that year.

## Results

The analysis was conducted by examining changes in strategies, time frame, complexity and expected outcome. As Table 1 shows, over the years, many programmes were introduced and different strategies employed. A list of all programmes by policy instrument is shown in Additional File 1.

**Table 1:** Programmes to address physician shortages in northern Ontario introduced by the Ontario government by policy instrument type and year, 1969 – 2004

	Financial incentive	Physician recruitment	Alternative providers	Rural medical education	Medical practice support	Service outreach	Travel assistance	Telehealth	Research
1969	**x x**		**x**			**x**			
1970		**x**							
1972				**x**					
1977					**x**				
1978		**x**							
1979	**x**				**x**				
1980									
1982					**x x**	**x**			
1985							**x**		
1991				**x**					
1992	**x**								**x**
1994					**x x**				
1995	**x**	**x**							
1996	**x x**			**x**					
1997	**x**				**x**				
1998		**x**			**x**			**x**	
1999	**x**			**x**		**x**			
2000	**x**		**x**	**x**					
2001	**x**								
2002		**x**		**x x x**	**x**				
2004		**x**							

Note: Each cross represents a programme. Multiple crosses in a cell indicate several programmes belonging to the same policy instrument type introduced in that year.

In the first two-and-a-half decades, new programmes were initiated at a relatively slow pace, at the rate of one or two a year. The exception was 1969, which was not surprising since the UAP was established in that year. There were periods spanning two to four years during which no new programmes were initiated. The speed of programme initiation picked up after 1995, sometimes with three to five programmes introduced in a year.

From the beginning, a variety of strategies were employed. In later years, the range of policy instruments used became even broader. For example, three new programmes were introduced in 2000, each representing a different policy tool. But financial incentive programmes were clearly the most often used. They came in different forms, ranging from bursaries for medical students to alternative funding schemes. Programmes to support medical practice, such as locum tenens programmes and virtual libraries, were also frequently used. So were physician recruitment programmes such as recruitment tours and the appointment of community development officers whose main job was to help northern communities find and keep doctors.

The first northern medical education initiative – the Northwestern Ontario Medical Program – started fairly early in 1972, though it was small in scale. The real investment in northern medical education occurred in 1991, when two family medicine residency programmes were established in Sudbury and Thunder Bay. The most significant initiative was the NOSM, the first medical school built in Canada in over 30 years.

Although many policy tools were employed, conspicuous by their absence were programmes of a directive nature, directive in the sense that physicians are required to work in northern or underserved communities for a certain period as a condition for admission to medical school or obtaining an OHIP billing number. Similarly, there were no programmes that sought to address spousal or family issues, which, as many studies have shown, are some of the most important factors in determining where physicians work.

Once introduced, a programme tended to persist. It might be modified, enriched or rolled into a new or bigger programme, but was rarely terminated. The few programmes that were discontinued include the Medical/Dental Centers Programme funded by the Ministry of Northern Development and Mines and the fee discounts measure, which penalized new physicians who chose to practise in "overserviced" areas by getting lower OHIP fee payments.

The programmes can also be analysed in terms of complexity in design and procedures. Some of the more recent programmes are more "sophisticated" in the sense that they tend to be more complex, more focused and better calibrated. For example, early financial incentive programmes were fairly simple, compared to more recent ones such as the Community Sponsored Contracts and Globally Funded Group Practices. Similarly, in terms of scale, complexity and ambition, the two family medicine residency programmes established in 1991 cannot be compared to the NOSM, which was inaugurated in 2005.

Another way to examine the programmes is in terms of the time frame of outcomes. Some programmes were designed to yield immediate results, while others were not expected to have an impact until years later. There is also the degree of outcome certainty. Some strategies or programmes are more "risky" in the sense that there is no certainty of tangible outcomes. For example, the Medical/Dental Centers Program was intended to quickly attract physicians and dentists by offering a "turnkey" facility at little or no cost to a physician or dentist willing to establish practice in the north. On the other hand, the NOSM can be seen as a long-term investment, since it will take many years before a student completes medical education and residency training and, even then, there is no guarantee that the new physician will work in northern Ontario. Similarly, the support of rural health workforce research is a long-term strategy, since research typically does not yield immediate results, but tends to focus on more complex or fundamental issues and explore innovative solutions. Programmes introduced in earlier years tended to have short- or medium-term time frames, while many of the programmes with a longer-term perspective were introduced in the 1990s and 2000s.

Efforts to overcome physician shortages can be divided into two major categories: recruitment and retention. Whereas the former is an effort to get a doctor to set up practice in a community, the latter is an attempt to keep the doctor there as long as possible. Recruitment without retention often results in a "revolving door" phenomenon – physicians come and go. While government efforts have focused mostly on recruitment, some programmes, such as the locum tenens programmes and alternative funding models, were designed with retention in mind. Overwork, burnout and a feeling of isolation are some of the factors leading to physicians' abandoning northern practice. Locum tenens programmes, for example, were intended to allow physicians in small communities to take time off work for holidays or continuing medical education. Similarly, by allowing physicians in remote places to keep up with the latest developments in the field, the Northern Ontario Virtual Library, which provided access to databases, journals and books via the Internet, could be seen as a means to reduce isolation. Programmes initiated in earlier years focused mostly on recruitment, whereas those intended to retain physicians came later. For instance, although there were many incentive programmes, the earlier ones were mostly for enticing physicians to work in the north by offering financial inducement. More recent programmes were mostly in the form of alternative payment schemes, which were designed to allow small-town doctors to opt out of fee-for-service payments, which tended to encourage doctors to see as many patients as possible, often resulting in overwork and burnout.

There does not appear to be a strong connection between provincial elections and programme initiation. During the study period, provincial elections in Ontario were held in 1971, 1975, 1977, 1981, 1985, 1987, 1990, 1995, 1999 and 2003. It is not apparent that many new programmes were introduced in election years as a way of garnering electoral support for the governing party (though it is possible that new initiatives were promised during election campaigns). In fact, a few election years saw no introduction of any new programme. There was a raft of new programmes introduced in 1999, but this may have more to do with the faster pace of programme initiation since 1996, as noted earlier, than with the provincial election.

There were several internal reviews of individual programmes, which resulted in some programme fine-tuning. For instance, the Northern Health Travel Grants Program was reviewed a couple of times and the Visiting Specialist Clinics Program was reviewed in 1999. In addition, there was a major review of the entire UAP in the early 1990s [34]. But it does not appear that the UAP was substantially changed in the years following this review. Another major review of the UAP took place in 2001–2003. The uncharacteristic lack of new programmes in 2003 and 2004 could be due to a wait-and-see attitude following the review.

## Discussion

Many questions have emerged from the above analysis. For instance, why were certain policy instruments used and not others? Why were there changes over the years?

### Changes over the years

The nature, or perceptions, of physician workforce issues changed over time. In the mid-1960s, just before the UAP debut, Canada was seen by the Royal Commission on Health Services as having doctor shortages. By the late 1980s and early 1990s, there was a belief – at least among governments – that Canada had a surfeit of physicians. This resulted in a number of measures to control physician supply. But, by the late 1990s, the pendulum swung back to the other side, as reflected by widespread concerns about physician shortages. Thus, building a medical school in the north would have been unthinkable in the early 1990s when Canadian medical schools were told to curtail enrolment. The mushrooming of new programmes in the late 1990s and the early 2000s may reflect growing unease about the need for physicians not just in the north, but also in some southern Ontario cities.

The late adoption of technology-related strategies is understandable. Telemedicine is a case in point. Although some form of telemedicine has existed ever since Alexander Graham Bell invented the telephone, its more widespread adoption has occurred only in the last decade or two when communications technologies have become more sophisticated, reliable and affordable. Similarly, the introduction of the Northern Ontario Virtual Library to support clinicians in far-flung places would not have been possible before the advent of the information technology age.

It is also apparent that attempts were made in more recent years to deal with fundamental issues and not just provide symptomatic relief. As noted earlier, financial incentive programmes in the early years were designed primarily to recruit doctors to work in northern areas. More recent incentive programmes began to address retention issues by allowing rural physicians to opt out of fee-for-service reimbursement. Similarly, the decision to build a new medical school was probably made after the realization that in the long run, the north needs to "grow" some of its own doctors, instead of relying totally on imports.

### Choice of policy instruments

Although many programmes were introduced over the years, interestingly, there were no programmes of a directive nature. It has been said that public policies are whatever governments choose to do or *not *to do [35]. Thus, it is important to know not just policy instruments that have been adopted, but also those not pursued. However, Brooks and Miljan [16] are right in pointing out that it makes no sense to talk about policy when an issue has not yet surfaced. "Once it has, however, inaction by policy-makers becomes a deliberate policy choice" (p. 5).

The absence of programmes of a directive nature is not because the issue and strategic alternatives have not surfaced. Some provinces, such as British Columbia [36], adopted or attempted to adopt measures whereby the issuance of physician billing numbers could be tied to geographical locations of practice as a way to channel physicians to underserved areas. In the mid-1990s, the Ontario government introduced Bill 26, *The Savings and Restructuring Act*, which contained provisions that allowed the Minister of Health to decide which areas of the province were "over-supplied" with physicians and to refuse issuing OHIP billing numbers to new physicians wishing to work in those areas. This was meant to direct new doctors to "under-supplied" areas. But the proposed measure was never implemented because of opposition by organized medicine, particularly the Professional Association of Internes and Residents of Ontario (PAIRO). Instead, PAIRO urged the use of alternative funding models and direct contracts, which, according to one estimate, "will generally entail a 20 per cent increase in pay" [37] (p. 41). It seems that the government has heard such messages loud and clear. This may also explain the discontinuation of the fee discounts measures, first introduced in 1996. Thus, the absence of directive measures can be seen as a deliberate choice of policy instrument.

The lack of programmes to address individual, spousal or family concerns is understandable. There is very little governments can do to alter lifestyle preferences, shape family relationships or satisfy spouses' career aspirations. Public policy may be too blunt an instrument to use for tackling problems that are highly personal or idiosyncratic in nature. This may be an area considered to be the realm of private behaviours not suitable for government intervention [31].

There were some travel-related programmes. This is not surprising, since "rural" and "northern" in the Canadian context typically imply vast territory and the concomitant need to travel. Access to care means either bringing services to people or people to services. The former include such programmes as the Visiting Specialist Clinics Program. An example of the latter is the Northern Health Travel Grants Program, which provides subsidies to patients who have to travel long distances to gain access to medical care. Another form of travel is telehealth. As Pong and Pitblado [38] have suggested, "telehealth can be seen as a form of mobility, involving long-distance 'travelling' by patients to see their physicians or vice versa by means of telecommunications" (p. 109).

The creation of the Northern Health Human Resources Research Unit (later renamed the Centre for Rural and Northern Health Research) in 1992 indicates a realization that policy and programme development should be evidence-based. It is also possible that the increasing reliance on the rural medical training strategy, including the building of a medical school in northern Ontario, has been influenced, at least in part, by research. The last two decades have witnessed a growing body of literature on the relationship between rural medical education and rural medical practice [9,39-41]. Studies conducted in Canada and elsewhere generally support the notion that doctors with an extensive rural exposure are more likely to practise in rural areas.

### Use of multiple strategies

Overcoming physician maldstribution is not an easy task. That several strategies were introduced at the outset suggests an early awareness that the problem was complex and could be dealt with only by using a variety of strategies. It is not just the adoption of multiple strategies but also the simultaneous use of different strategies that is worth noting. The policy implementation process does not appear to be sequential in the sense that a programme or strategy became outdated and was replaced by a new one. Instead, few programmes were ever terminated. Also, some strategies were used over and over in the form of programmes with different names but of a similar nature: witness the number of locum tenens programmes and alternative payment models. The use of multiple strategies is especially evident in more recent years as many older programmes were retained and new ones added.

It is not known why strategies and programmes were used simultaneously. Could it be that once a programme has been introduced, it creates a constituency that ensures its continuation? Is it because the existence of many programmes gives the impression of government attention and action? Or is it because policy-makers have seen the need for a bundling of several policy instruments as a response to complex problems? Apparently physician maldistribution is not like a disease that can be cured, but is more akin to a chronic condition that needs to be managed. Extending the medical analogy further, one could liken the use of multiple strategies to polypharmacy, with the potential danger of drug interaction. Tepper and associates [28] have made a similar diagnosis when they highlight the use of many policy initiatives and point out that "(t)he amount of overlap also raises the question as to whether a more integrated approach to policy planning would be helpful" (p. 33).

### Limitations

This study has some limitations. First, it has treated every programme or strategy as equal. This clearly is not true. Some programmes are considerably more complex and costly, and presumably have a greater impact, than others. Second, the study has focused on the initiation of programmes and disregarded changes following programme introduction. But such changes have tended to be administrative in nature and do not indicate strategic redirection. Third, this study has not included physician workforce initiatives that do not have a specific northern Ontario focus. Some of these "generic" measures, such as expansion of medical school enrolment and allowing more international medical graduates to practise, may have a bigger impact on northern Ontario than some of the northern-specific programmes. However, their effects on the north have not been empirically determined or documented to date.

## Conclusion

This study has looked at a policy implementation process that has spanned 35 years. Although the policy goal of increasing physician supply and ensuring better distribution in northern Ontario has remained the same, the strategies and programmes used to implement the policy have evolved over time. This suggests that an examination of a policy is incomplete if implementation has not been taken into consideration. The nature of a policy is delineated, if not determined, by how it is put into action. In this sense, Dye's [35] notion of policy as what a government chooses to *do *or not to *do *is an insightful one. It is the "do" aspect in the policy process that gives meaning and substance to policies.

Studies of policy implementation, as well as evaluation, typically focus on a single policy instrument or programme over a relatively short period. Such studies, while useful in shedding light on the nature of a programme or the efficacy of a strategy, often fail to reveal the trajectories of policy implementation. This study has shown that a longer-term perspective is needed, because while a policy may remain more or less the same, its implementation and the instruments used may evolve over time in response to changing circumstances.

In addition, this study has shown that from a policy implementation perspective, rational and incremental processes are not necessarily mutually exclusive. It has revealed a process of trial-and-error experimentation and an accumulation of past experience. By examining when programmes were introduced and what policy tools were adopted over 35 years, the study has shown that programmes introduced more recently tend to be more complex, are more likely to take a longer time perspective, and pay more attention to physician retention.

But the choice of policy instruments is not just a function of what Sabatier [18] calls policy-oriented learning. It may also be constrained by objective conditions or perceptions of reality. Although many policy tools may have been contemplated, there appear to be limits to what can be adopted. Strategies and programmes opposed by powerful vested interests, such as measures seen as "coercive" by physicians, have mostly fallen by the wayside. This may explain the heavy reliance on financial incentives, recruitment programmes and rural medical training. Thus, policy implementation represents the compromise or accommodation that often eventuates when policy meets reality.

This study has shown how the problem of physician shortages in northern Ontario has been addressed by the provincial government over a 35-year period. The programmes and strategies documented are revealing in and of themselves. Equally important, they shed light on the intricate relationships between policy, policy implementation and the use of policy instruments and programmes.

## Abbreviations

NOSM: Northern Ontario School of Medicine; OHIP: Ontario Health Insurance Plan; PAIRO: Professional Association of Internes and Residents of Ontario; UAP: Underserviced Area Program.

## Competing interests

The author declares that they have no competing interests.

## Supplementary Material

Additional file 1 **Chronology of provincial government programmes to address physician shortages in northern Ontario, 1969–2004.** Click here for file
