# Supplementary material for: Strategies to overcome physician shortages in northern Ontario: A study of policy implementation over 35 years
Source: Hum Resour Health. 2008 Nov 11;6:24. doi: 10.1186/1478-4491-6-24 (PMC2588636; doi:10.1186/1478-4491-6-24)
Supplement: Additional file 1 — Chronology of provincial government programmes to address physician shortages in northern Ontario, 1969–2004. [file 1478-4491-6-24-S1.doc]

### Additional File 1. Chronology of provincial government programmes to address physician shortages in northern Ontario, 1969–2004

| **Year of programme initiation** | **Name of programme** | **Policy instrument type** |
| --- | --- | --- |
| 1969 | UAP Incentive grants | Incentive |
| 1969 | Remote nursing stations | Alternative Provider |
| 1969 | Physician Outreach Program | Outreach |
| 1969 | Northern Bursary Program | Incentive |
| 1970 | Community Assessment Visit Program | Recruitment |
| 1972 | Northwestern Ontario Medical Program | Rural training |
| 1977 | Medical/Dental Centers Program | Practice support |
| 1978 | Health Professionals Recruitment Tour | Recruitment |
| 1979 | Respite Locum Program | Practice support |
| 1979 | Basic and Northern Medical Special Incentive Programs | Incentive |
| 1982 | Visiting Specialist Clinic Program | Outreach |
| 1982 | Urgent Locum Program for Specialists | Practice support |
| 1982 | Respite Locum Program for Specialists | Practice support |
| 1985 | Northern Health Travel Grants Program | Travel assistance |
| 1991 | Northeastern and Northwestern Family Medicine Residency Programs | Rural training |
| 1992 | Service Retention Initiative | Incentive |
| 1992 | Northern Health Human Resources Research Unit | Research |
| 1994 | Ontario Medical Association Continuing Medical Education Program for Rural and Isolated Physicians | Practice support |
| 1994 | Ontario Medical Association Rural Locum Program | Practice support |
| 1995 | Sessional Fee for Rural Emergence Departments | Incentive |
| 1995 | Community Development Officers | Recruitment |
| 1996 | Re-entry Program | Rural training |
| 1996 | Community Sponsored Contracts | Incentive |
| 1996 | Fee discounts | Incentive |
| 1997 | Globally Funded Group Practices | Incentive |
| 1997 | Medical Services Corp | Practice support |
| 1998 | Ontario Medical Association Physician Job Registry | Recruitment |
| 1998 | Ontario Medical Association Respite Locum Program for General Practitioners/Family Physicians in Rural and Isolated Areas | Practice support |
| 1998 | NORTH Network | Telemedicine |
| 1999 | Northern Academic Health Sciences Network | Rural training |
| 1999 | Ontario Psychiatric Outreach Program | Outreach |
| 1999 | Alternative Funding Agreements for rural EDs | Incentive |
| 2000 | Nurse practitioner positions for northern Ontario | Alternative providers |
| 2000 | Free Tuition Program | Incentive |
| 2000 | Northeastern Ontario Post-graduate Specialty Program | Rural training |
| 2001 | Northern Physician Retention Initiative | Incentive |
| 2002 | Northern Ontario School of Medicine | Rural training |
| 2002 | Rural and Northern Clerkship | Rural training |
| 2002 | Northern Ontario Virtual Library | Practice support |
| 2002 | Northeastern Stream Residency Program | Rural training |
| 2002 | Professional Association of Internes and Residents of Ontario Registry | Recruitment |
| 2004 | Professional Association of Internes and Residents of Ontario Resident Placement Program | Recruitment |
